# The Global Emergency of Novel Coronavirus (SARS-CoV-2): An Update of the Current Status and Forecasting

**DOI:** 10.3390/ijerph17165648

**Published:** 2020-08-05

**Authors:** Hossein Hozhabri, Francesca Piceci Sparascio, Hamidreza Sohrabi, Leila Mousavifar, René Roy, Daniela Scribano, Alessandro De Luca, Cecilia Ambrosi, Meysam Sarshar

**Affiliations:** 1 Department of Experimental Medicine, Sapienza University of Rome, 00161 Rome, Italy; hossein.hozhabri@uniroma1.it (H.H.); francesca.piceci@uniroma1.it (F.P.S.); 2 Medical Genetics Division, Fondazione IRCCS Casa Sollievo della Sofferenza, 71013 San Giovanni Rotondo, Italy; a.deluca@css-mendel.it; 3 Department of Veterinary Science, University of Turin, 10095 Grugliasco, Italy; hamidreza.sohrabi@edu.unito.it; 4 Department of Chemistry, Université du Québec à Montréal, P.O. Box 8888, Succ. Centre-Ville, Montréal, QC H3C 3P8, Canada; mousavifar.seyedehleila@uqam.ca (L.M.); roy.rene@uqam.ca (R.R.); 5 INRS-Institut Armand-Frappier, Université du Québec, 531 boul. des Prairies, Laval, QC H7V 1B7, Canada; 6 Department of Public Health and Infectious Diseases, Sapienza University of Rome, 00185 Rome, Italy; 7 Dani Di Giò Foundation-Onlus, 00193 Rome, Italy; 8 IRCCS San Raffaele Pisana, Department of Human Sciences and Promotion of the Quality of Life, San Raffaele Roma Open University, 00166 Rome, Italy; cecilia.ambrosi@uniroma5.it; 9 Department of Public Health and Infectious Diseases, Sapienza University of Rome, Laboratory affiliated to Institute Pasteur Italia- Cenci Bolognetti Foundation, 00185 Rome, Italy; 10 Research Laboratories, Bambino Gesù Children’s Hospital, IRCCS, 00146 Rome, Italy; 11 Microbiology Research Center (MRC), Pasteur Institute of Iran, 1316943551 Tehran, Iran

**Keywords:** SARS-CoV-2, epidemiology, transmission, temperature and humidity, COVID-19, pneumonia, diagnosis, ACE2, inhibitors, therapeutics strategies

## Abstract

Over the past two decades, there have been two major outbreaks where the crossover of animal *Betacoronaviruses* to humans has resulted in severe acute respiratory syndrome coronavirus (SARS-CoV) and Middle East respiratory syndrome coronavirus (MERS-CoV). In December 2019, a global public health concern started with the emergence of a new strain of coronavirus (SARS-CoV-2 or 2019 novel coronavirus, 2019-nCoV) which has rapidly spread all over the world from its origin in Wuhan, China. SARS-CoV-2 belongs to the *Betacoronavirus* genus, which includes human SARS-CoV, MERS and two other human coronaviruses (HCoVs), HCoV-OC43 and HCoV-HKU1. The fatality rate of SARS-CoV-2 is lower than the two previous coronavirus epidemics, but it is faster spreading and the large number of infected people with severe viral pneumonia and respiratory illness, showed SARS-CoV-2 to be highly contagious. Based on the current published evidence, herein we summarize the origin, genetics, epidemiology, clinical manifestations, preventions, diagnosis and up to date treatments of SARS-CoV-2 infections in comparison with those caused by SARS-CoV and MERS-CoV. Moreover, the possible impact of weather conditions on the transmission of SARS-CoV-2 is also discussed. Therefore, the aim of the present review is to reconsider the two previous pandemics and provide a reference for future studies as well as therapeutic approaches.

## 1. Introduction

### 1.1. What Are Coronaviruses

Coronaviruses (CoVs) are a group of highly enveloped viruses that are diversely found in humans and wildlife. With their high mutation rate and infectivity, CoVs are important zoonotic pathogens that can infect animals [1,2] and humans, leading to 5–10% of acute respiratory syndromes [3]. Apart from infecting a variety of economically important vertebrates (such as pigs and chickens), six species have been identified to cause disease in humans [4]. They are known to infect respiratory, gastrointestinal, hepatic and neurologic systems with a wide range of clinical features from asymptomatic course to severe disease that require hospitalization in the intensive care unit [4,5]. The first human coronaviruses (HCoVs), HCoV-229E and OC43, shown to be significant respiratory pathogens, were identified in the 1960s [6,7]. However, it is assumed that the first recorded coronavirus-related disease was feline infectious peritonitis (FIP) in 1912 [8]. The “corona”-like or crown-like morphology of these viruses leads to choose the name “coronavirus,” in 1968 [6].

Coronaviruses were not considered as highly pathogenic for humans before the beginning of the 21st century. Afterward, two highly pathogenic HCoVs, including severe acute respiratory syndrome coronavirus (SARS-CoV) and Middle East respiratory syndrome coronavirus (MERS-CoV), emerging from animal reservoirs, have led to global epidemics of deadly pneumonia in humans with high morbidity and mortality [9,10]. In December 2019, seven years after MERS outbreak, the third pathogenic HCoV emerged in Wuhan, the capital city of Hubei province in China, causing severe pneumonia [11,12]. Considered as agents that are a great public health threat, an epidemiological alert was placed by the World Health Organization (WHO) and this new coronavirus was named SARS-CoV-2 and the related respiratory disease COVID-19 (https://www.who.int). Compared with SARS-CoV, SARS-CoV-2 appears to be more readily transmitted from human-to-human, spreading to multiple continents and the outbreak of SARS-CoV-2 was declared on January 30, 2020 [13] (https://www.who.int). In this review, we will introduce the current knowledge on the origin and evolution of SARS-CoV-2, emphasizing its characteristics and its genetic diversity from previous coronaviruses, with a brief comment on its epidemiology and pathogenesis. We also highlight the environmental factors involved in virus transmission. Knowledge about this novel coronavirus is rapidly evolving, and efforts must be implemented in order to protect the populations by reducing transmission and controlling the spread of this fatal disease.

### 1.2. Origin, Family Member, Diversity and Taxonomy of Coronaviruses

**Figure 1:**
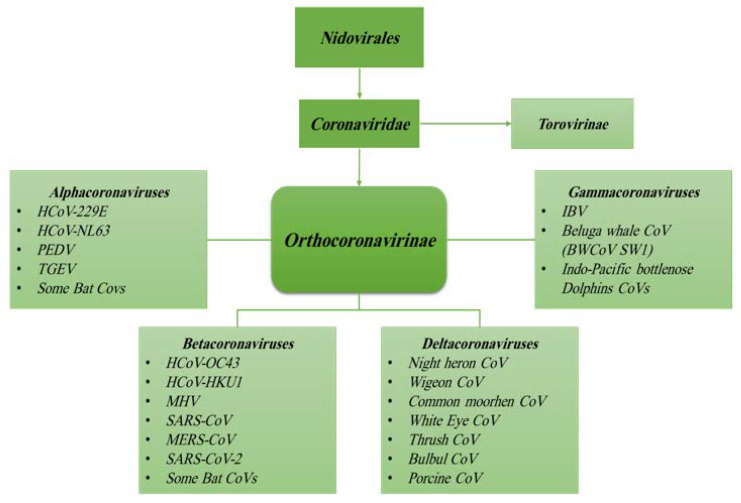
Classic subgroup clusters of coronaviruses within the family *Coronaviridae*, subfamily *Orthocoronavirinae* and the respective genera: *Alphacoronavirus*, *Betacoronavirus*, *Gammacoronavirus* and *Deltacoronavirus*.

According to the International Committee on Taxonomy of Viruses, CoVs are classified under the order of *Nidovirales*, a family of *Coronaviridae* and subfamily of *Coronavirinae* [14]. Based on previous serologic and recent genomic evidences, the family of *Coronaviridae* encompasses two subfamilies: subfamily *Orthocoronavirinae* and subfamily *Torovirinae* (Figure 1) [7,15]. The subfamily of *Orthocoronavirinae* consists of four genera: *Alphacoronavirus*, *Betacoronavirus*, *Gammacoronavirus* and *Deltacoronavirus* [7,16,17].

**Figure 2:**
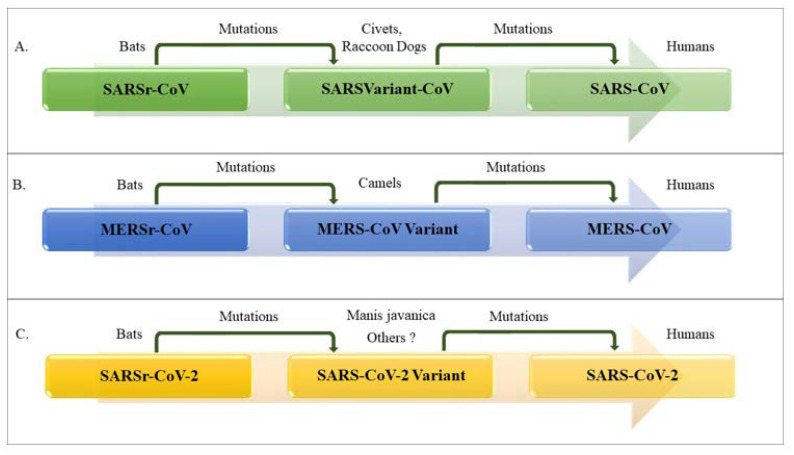
Origin and evolution of (**A**) SARS-CoV, (**B**) MERS-CoV and (**C**) SARS-CoV-2 in the various hosts. Initially all viruses existed in diverse bat species as CoV-related viruses (SARSr-CoV, MERSr-CoV and SARSr-CoV-2); sequential mutations and recombinations allow them to adapt to intermediate hosts and finally humans [15].

CoVs can be isolated from different animal species, including birds, livestock and mammals such as camels, bats, masked palm civets, mice, dogs and cats [18,19]. Animal CoVs are known to cause acute diseases in several animals and could be responsible for economic losses in domestic animals or birds [20,21]. Domestic animals may play an important role as intermediate hosts that enable virus transmission from one species to humans [17]. The genera *Gamma*- and *Deltacoronavirus* infect birds, but some of them can also infect mammals [16]. These animal CoVs include transmissible gastroenteritis virus (TGEV), porcine epidemic diarrhea virus (PEDV), avian infectious bronchitis virus (IBV)—and more recently—swine acute diarrhea syndrome coronavirus (SADS-CoV). However, animal CoVs can also infect humans that can spread the infection through human-to-human transmission [17,22]. On the other hand, *Alpha*- and *Betacoronavirus* infect only mammals and usually cause respiratory illness in humans; among these, strains 229E, OC43, HKU1 and NL63 are the most widespread infecting young children, infants as well as elderly individuals [23,24,25]. The high rates of mutation characterizing all RNA viruses [23,26], the evolving nature of CoVs and the simplicity of transmission from one species to another are the most relevant features learned from SARS-CoV and MERS-CoV previous outbreaks [15,23,25]. Importantly, most of *Alpha*- and *Betacoronavirus* were found only in bats, and many genetically diverse coronaviruses phylogenetically related to SARS-CoV and MERS-CoV have been discovered in diverse bat species worldwide [17]. Therefore, HCoVs such as SARS- and MERS-CoVs seem to have originated in bats by sequential mutations and recombination, including those occurring in the intermediate hosts, civets and raccoon dogs for SARS-CoV and camels in the case of MERS-CoV, finally acquiring the ability to infect humans [15,17]. Comparative genome studies published in recent papers strongly support the hypothesis that SARS-CoV-2 originated in bats and that pangolins (Manis javanica) acted as intermediate mammalian hosts [11,27] (Figure 2). Indeed, the genetic sequence of the SARS-CoV-2 showed more than 79% nucleotide identity with the sequence of SARS-CoV and 50% with MERS-CoV [17,19]. The high degree of homology of the angiotensin-converting enzyme 2 (ACE2) receptor in several animal species can be considered as an additional evidence to support that SARS-CoV-2 originated from bats [28]. Based on findings from molecular studies, the ACE2 proteins of non-human primates, pigs, cats and ferrets closely resemble the human ACE2 receptor. Therefore, these species may be susceptible to SARS-CoV-2 infection, as has been shown for SARS-CoV. Although a recent study showed that neither pigs nor chickens are susceptible to SARS-CoV-2 by intranasal or oculo-oronasal infections, more evidences are needed to exclude pigs as intermediate host of SARS-CoV-2 [29].

Based on the genetic sequence identity and the phylogenetic reports, SARS-CoV-2 is sufficiently different from SARS-CoV; thus, WHO has classified it as a new *Betacoronavirus* that infects humans [30].

### 1.3. Morphology and Genomic Structure of HCoVs

**Figure 3:**
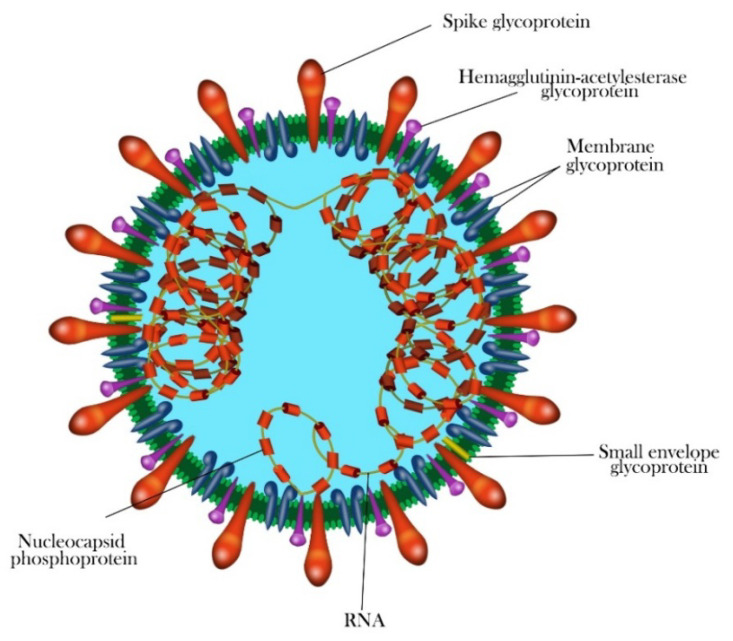
Typical coronavirus virion structure and proteins. The coronavirus genome encodes a (S) spike glycoprotein, an (E) envelope glycoprotein, a (M) membrane glycoprotein, a (N) nucleocapsid phosphoprotein and a (HE) hemagglutinin-esterase glycoprotein.

**Figure 4:**
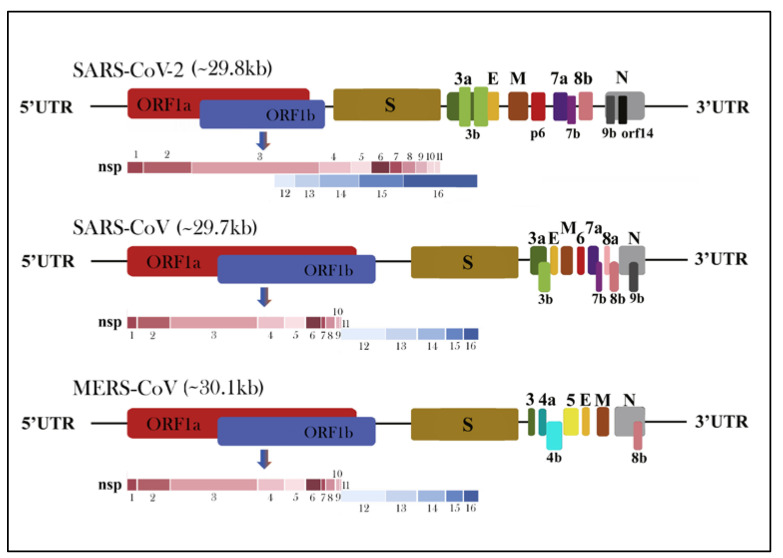
Graphic genome structures of SARS-CoV-2, SARS-CoV and MERS-CoV. Each coronavirus (CoV) genome is schematically represented in the order of 5′-ORF1a-ORF1b-S-E-M-N-3′. The coronavirus genomes encode two replicase polypeptides pp1a and pp1ab translated from ORF1a and ORF1b; four structural genes encoding for four structural proteins including (S) spike, (M) membrane, (E) envelope and (N) nucleocapsid proteins. The single-stranded RNA genomes of SARS-CoV-2 (~29.8 kb), SARS-CoV (~29.7 kb) and MERS-CoV (~30.1 kb) harbor two large genes, the ORF1a (red) and 1b (blue) genes encoding accessory genes (nsps 1–16, shades of red and blue). Encoded nonstructural proteins: 16 nsps (nsp1-nsp16) in SARS-CoV-2, SARS-CoV and MERS-CoV. Along with structural proteins (S, E, M and N), the 3′-terminus of the SARS-CoV-2 and SARS-CoV genomes contain eight accessory proteins (3a, 3b, p6, 7a, 7b, 8b, 9b and orf14 and 3a, 3b, p6, 7a, 7b, 8a, 8b and 9b, respectively) while MERS-CoV genome contains only five (3, 4a, 4b, 5 and 8b). The genes encoding accessory proteins are unique in different coronaviruses in terms of number, genomic organization, sequence and functions (data extracted from [35,49,57]).

The genome of HCoVs is a single-stranded positive-sense RNA (+ssRNA) (~26–32 kb) with 5′-cap structure and 3′-poly A tail, which is among the largest known RNA genomes [31,32,33]. The typical HCoVs gene order is 5′-replicase-S-E-M-N-3′, with numerous (6 to 11) open reading frames (ORFs) encoding accessory proteins scattered among the structural genes [34,35]. The first ORFs (ORF1a and 1b) comprise two-thirds (approximately 67%) of the genome length and encode 16 nonstructural polyproteins (nsps 1–16) and are directly translated from the genomic RNA [17]. There is a −1 ribosomal frameshift between ORF1a and ORF1b, leading to the production of two large replicase polypeptides (pp): pp1a and pp1ab. These polypeptides are further processed by two virally encoded cysteine proteases, the papain-like protease (PLpro) and a 3-chymotrypsin-like protease (3CLpro) into 16 nsps [3,33,36]. There are at least four structural proteins encoded by the coronaviral genome: a spike glycoprotein (S), an envelope protein (E), a membrane protein (M) and nucleocapsid protein (N) with short untranslated regions at both termini, required to produce a structurally complete viral particle [37]. The typical coronavirus virion structure and proteins are shown in Figure 3. The M protein is in higher quantities in comparison to any other proteins in the virus particle; with its three transmembrane domains, it shapes virions, promotes membrane curvature and binds to the nucleocapsid [38,39]. The N protein contains two domains, both of which can bind to nsp3 protein to help tether the genome to replication–transcription complex (RTC) and package viral RNA into the viral particle during viral assembly [39,40]. The E protein is involved in virus assembly and virion release from host cells, while the S protein plays a vital role in attachment to host receptors, viral entry and determines host tropism [41,42]. Additionally, some coronaviruses, such as HCoV-OC43 and HCoV-HKU1, have a hemagglutinin-esterase (HE) gene between ORF1b and S [43,44,45,46]. This hemagglutinin, like the influenza homolog enzyme, binds to sialic acid on host cell-surface glycoproteins and possesses acetyl-esterase activity [47]. Besides coronavirus-conserved genes, the SARS-CoV, SARS-CoV-2 and MERS-CoV genomes contain several specific accessory genes including ORF3a/b, 4a/b, ORF5, ORF6, ORF7a/b, ORF8a/b and 9b (Figure 4) [4,48,49]. All the structural and accessory proteins are translated from subgenomic RNAs (sgRNAs) generated during genome transcription/replication of CoVs [4].

### 1.4. Attachment, Entry and Life Cycle of HCoVs

Attachment, cell entry, translation of viral replicase, genome replication, translation of structural proteins and virion assembly and release are the phases of coronavirus replication cycle [4,50]. SARS-CoV, MERS-CoV and SARS-CoV-2 bind to different host receptors to gain entry into host cells [4,51,52]. Viral entry is mediated by the transmembrane S glycoprotein that comprises two functional subunits (S1 and S2 subunits) responsible for receptor recognition and viral-host cell membranes fusion, respectively [53,54]. S1 receptor-binding domain (RBD) mediates binding to the cognate host cell receptor; however, the S2 domain mediates the fusion events, between viral envelope and host cell membrane [52,55,56]. As recently found, SARS-CoV-2 uses the same ACE2 receptor [57], as SARS-CoV, whereas MERS-CoV uses dipeptidyl peptidase 4 (DPP4, also known as CD26) receptor (Table 1) [58]. The fusion of the S protein to the plasma membrane of host cell generates a double membrane vesicle in the host cell, thereby allowing release of the nucleocapsid into the cytoplasm, followed by genome transcription [53,54]. Upon entry into the cell, virus-specific RNA and proteins are synthesized, probably entirely in the cytoplasm. Translation starts with the expression of two polyproteins, pp1a and pp1ab, which undergo co-translational proteolytic processing into the proteins that form the replicase complex. This complex is used to transcribe a 3′-coterminal set of nested subgenomic mRNAs, as well as genomic RNA that have a common 5′ “leader” sequence derived from the 5′ end of the genome. New virions are assembled by budding into intracellular membranes of the pre-Golgi compartment and released through the cell secretory mechanisms [4,42,48,50].

## 2. Pathogenesis and Mode of Transmission

Virologic as well as genetic studies have demonstrated that bats are reservoir hosts of both SARS-CoV and MERS-CoV, but also that they can use other species as intermediate hosts before spreading to humans [59,60]. The detection of two genomes distinct from known swine in ill piglets were reported by two independent groups [61,62]. The phylogenetic analyses showed that these novel swine enteric *Alphacoronaviruses* (SeACoVs) were strongly related to the *Rhinolophus* bat coronavirus HKU2 isolated in Guangdong Province, in southern China [61,62]. This suggests that coronaviruses of bat origin may have ‘jumped’ the barrier of the species to infect pigs as intermediate hosts. The CD26 receptor sequence alignment between humans and pigs demonstrates a 94.5% overlap, which is sufficient for the possible cross-species transmission [63]. It has also been documented that pigs are susceptible to human SARS-CoV [64] and MERS-CoV infections [65]. The large number of mutations within the RBD enabled viruses to infect new hosts, representing a potential threat for both animal and human health. In southern China, the unique climate, the high density of domestic as well as wild pigs, along with the extensive bat distribution and carriage of tremendous quantities of recombinant novel coronaviruses may result in the appearance of more novel coronaviruses in the future [66]. It is generally acknowledged that numerous viruses have existed and were restricted to their natural reservoirs for lengthy times [17]. The consistent spillover of viruses from natural hosts to humans and other species is essentially related to human activities, including urbanization and modern agricultural practices, leading to the constant human exposure to the ever-changing mutant CoVs from their reservoirs [15,17]. The close contact between humans and animals and the practice of eating raw meat are both risk factors for causing a new human CoV outbreak [15]. Hence, COVID-19 should be considered as a zoonotic disease that spread from animals to humans.

Following the first SARS-CoV-2 outbreak in seafood and wildlife market in Wuhan, secondary cases started to be identified after ten days. Although these new patients did not have any contact with the market, they had a history of contact with people who attended the market [60]. Therefore, similarly to SARS-CoV and unlikely to MERS-CoV, human-to-human transmission for SARS-CoV-2 has been reported and is currently considered as the main type of transmission worldwide [5,19]. On January 13, 2020, Thailand announced the first non-Chinese case of infection that spread from the Chinese provinces, to the Asian continent [60]. This case was a Chinese tourist who has traveled to Thailand and did not have any epidemiological link to the market [30]. More recently, Forster et al., by using phylogenetic analysis based on nucleotide mutations of 160 complete human SARS-CoV-2 genomes found that three variants of SARS-CoV-2 (A as the ancestral type, plus B and C) represent the bat outgroup coronaviruses. In particular, the A and C types were found mostly in European- American patients, whereas the B type was common in East Asia suggesting that this kind of analysis could help in following the evolution of SARS-CoV-2 [67].

### Human-to-Human Transmission and Viral Reservoir

It was demonstrated that SARS-CoVs have adapted themselves to bind to human ACE2 receptor and infect human cells effectively [68]. This form of adaptation required a series of amino acid changes in the RBD within the S protein of SARS viruses that circulated in bats [56,68]. Therefore, the human-to-human transmission that was seen in the course of the SARS-CoV outbreaks is directly attributable to the ability of SARS-CoVs to adapt their S protein to efficiently bind to human ACE2, thus infecting ciliated bronchial epithelial cells and type II pneumocytes [15,69]. Similar to SARS-CoV, ACE2 is also used by SARS-CoV-2 as the entry receptor in the ACE2-expressing cells, suggesting that SARS-CoV-2 has a life cycle similar to SARS-CoV [56,68]. As outlined before, SARS-CoV S protein regulates the receptor binding and membrane fusion activities determining host tropism and transmission capacity. Several evidences highlighted a higher binding affinity of SARS-CoV-2 RBD to the ACE2 receptor. In particular, molecular and in silico analyses demonstrated that SARS-CoV-2 RBD conformation and amino acid composition enhance the bonding between the S protein and ACE2 receptor [51,70,71]. A recent biophysical and structural analysis of the SARS-CoV-2 S protein showed that it binds to ACE2 receptor with about 10- to 20-fold higher affinity than the S protein of SARS-CoV [52,72]. This high affinity could account for its extreme infectivity among human populations. Another feature of the powerful infectivity of SARS-CoV-2 is that the shedding pattern of viral particles in SARS-CoV-2 diagnosed patients is similar to that of influenza patients in which viral loads at the time of symptom onset are higher and gradually decrease within days; interestingly, this pattern seems to be different from that reported for SARS-CoV patients where the highest shedding is reported 10 days after the onset of symptoms [20,73,74]. These results indicate that SARS-CoV-2 can spread more easily than SARS-CoV in the community even in the absence of symptoms or when only initial mild symptoms are present [75].

The human-to-human transmission of SARS-CoV-2 mainly occurs by inhalation of respiratory droplets spread by coughing or sneezing from an infected individual, but also by direct contact of contaminated surfaces and then touching the nose, mouth and eyes [24,76,77,78]. The virus was shown to remain stable in favorable atmospheric conditions on different surfaces for days [79]. Additionally, transmission in an unventilated environment or closed spaces due to high aerosol concentrations has been suggested [76,77]. In agreement, the presence of SARS-CoV-2 in the surfaces of the houses of confirmed patients was reported, further strengthening this mode of contact transmission. Moreover, live viruses were also found in the stool of COVID-19 patients, as previously found for both SARS-CoV and MERS-CoV [77]. Given its capacity for survival in feces and the expression of ACE2 within intestine, it was demonstrated that SARS-CoV-2 can infect these tissues and can be released in feces; therefore, water supply contamination and fecal-oral route transmission is also hypothesized [24,80]. However, at present, there have not been reported cases of fecal-oral transmission of the virus. Studies have also indicated that SARS-CoV-2 transmission via ocular surfaces should not be overlooked, as contaminated droplets and body fluids could easily infect the human conjunctival epithelium [81]. SARS-CoV-2 is also responsible for cluster transmission, in particular within family clusters [77]. In some cities, 50% to 80% of all reported cases of COVID-19 accounted for cluster transmission [82]. Based on the current information, there is no evidence for transplacental transmission from infected pregnant women to their fetus, who underwent caesarean section [24,78]. Therefore, whether transmission during vaginal birth can occur remains to be established, neonatal COVID-19 disease as postnatal transmission was documented [83]. Although, SARS-CoV-2 may definitely infect infants, it has been reported that neonates, infants and children develop significantly milder forms of the disease than their adult counterparts [24,84].

## 3. Epidemiology

Coronaviruses are responsible for 5–10% of acute respiratory illness while it has been estimated that 2% of the population is deemed as an asymptomatic carrier of these viruses. The first discovered HCoV was IBV that causes respiratory disease in human whereas, HCoV-229E and HCoV-OC43, which cause the common cold in humans [15,26,85]. They were not considered to be highly pathogenic for humans until the outbreak of SARS in Guangdong state of China in 2002 and 2003. SARS-CoV infected more than 8000 people worldwide and caused 916 deaths (Table 2), representing a mortality rate by around 10% [86]. Ten years later in 2012, MERS-CoV emerged in Saudi Arabia and infected over 2494 people with 858 deaths, accounting for a mortality rate approximately of 35% [9,24,87]. Starting in China in December 2019, there were reports of patients presenting severe viral pneumonia [15,88,89]. This public health concern resulted in many unknown pneumonia cases who were admitted to local hospitals [22,78] (https://www.who.int). Primary etiologic investigations performed in those patients showed that they were epidemiologically linked to a Huanan wholesale seafood market that also traded live animals and wildlife [17,24,90]. By January 7th, 2020 Chinese authorities announced that a new type of coronavirus was isolated [60,91]. The epicenter of infection was probably linked to a zoonotic pathogen being present in the seafood and exotic animal wholesale market [60,91]. The rapid increasing numbers and rate of fatalities indicated a second mode of transmission, from human-to-human, that allowed viral spreading primarily in other Asian countries such as South Korea and Iran followed by many countries such as Italy, Spain, Germany, France, Brazil and USA [24,60]. It is very intriguing to note that the SARS epidemic in southern China in 2002 and the current outbreak of COVID-19 had peaked in mid-February due to exposure to live animals sold in markets. Furthermore, similar to the SARS outbreak, this epidemic has occurred during the Spring Festival in China, as the most famous traditional countrywide festival in China, gathering nearly three billion people from different areas. These favorable conditions caused the wide transmission of this fatal pneumonia and severe difficulties for disease control and prevention of the epidemic [92].

Based on clinical data of diagnosed patients during the SARS-CoV-2 outbreak, the basic reproduction number (R0) is estimated to range between 2 and 6.47 in various modeling studies [76,93]. The SARS-CoV-2 R0 is in line with the one estimated for SARS-CoVs and MERS-CoVs (from 2 to 5) [94,95]. Currently, increasing countries are experiencing clusters of cases and community transmission following SARS-CoV-2 pandemic. Since its emergence, the COVID-19 has drawn well-deserved attention from authorities in order to protect their community and stop or slow down transmission of this disease. At the time of this review, according to the daily report of the World Health Organization, SARS-CoV-2 has affected over 17,889,134 people with around 228,611 daily new cases and killed more than 686,145 people all over the world, by August 3rd, 2020 (the up to date fatality rate is reported from https://covid19.who.int). We must take into consideration that these data are relative to laboratories and clinically confirmed cases while the actual number including asymptomatic cases, infected undiagnosed and death patients would be much higher than reported cases.

### The Possible Effects of Weather Conditions on the Transmission of SARS-CoV-2

The transmission of seasonal respiratory coronaviruses can be affected by several climate parameters such as temperature and humidity [96]. Therefore, understanding the relationship between weather and transmission of COVID-19 is the key to forecast the intensity and end time of this pandemic. To this regard, emerging evidence suggests that whether climate conditions may influence the transmission of the SARS-CoV-2 by boosting the spread (much of the data have not been peer-reviewed yet). To date, COVID-19 has had a significant expansion in the Northern Hemisphere (NH) belt, given that it covers cities and populated areas; conversely, in the belt of the Southern Hemisphere (SH), which covers very low population and landless areas, COVID-19 has not been reported yet.

Based on climate and ERA-interim reanalysis dataset in NH belt from November 2019 to March 2020, we compared the average rate of humidity and temperature between five cities in European Countries with significant community transmission of COVID-19 versus five cities of North Africa which are expected to be less exposed to COVID-19. The information recorded by the meteorological stations has been used, since these are more accurate than satellite data [97]. As shown in Table 3, the average amount of humidity is very close between European and African selected sites. The main reason is the proximity of these cities to the Mediterranean Sea coastline. In addition, the north wind, which blows from northern Europe to European cities (ECs), increases the humidity of these cities. Conversely, there are temperature differences between considered ECs and North African cities (Table 3). Thus, temperature and humidity should be considered parameters involved in the transmission of COVID-19. Up to know, few studies have investigated the association of temperature and humidity with COVID-19 incidence and death rates. The first meteorological study was done in 100 different Chinese cities each having more than 40 cases of COVID-19 in a 3-day period during the end of January [98]. This group showed that high temperature and humidity significantly reduces the transmission of COVID-19. Their results indicate that the increases of 1 °C in temperature and 1% in relative humidity lower R by 0.0225 and 0.0158, respectively [98]. A preprint study on confirmed COVID-19 cases collected from 429 cities showed that every 1 °C increase in the minimum temperature of higher-temperature cities reduced the disease incidence and death rates by 0.86 [99]. Another preprint study suggested that the average increase of 1 °C in temperature correlates negatively with the predicted number of cases worldwide [100]. These results are in accordance with Wu Y et al. who showed that among all confirmed COVID-19 new cases and new deaths from 166 countries (excluding China), a 1 °C increase in temperature is associated with a 3.08% reduction in daily new cases and a 1.19% reduction in daily new deaths, whereas a 1% increase in relative humidity was associated with a 0.85% reduction in daily new cases and a 0.51% reduction in daily new deaths [101]. A recent study conducted in Italy showed a positive correlation of SARS-CoV-2 spreading and weather conditions including temperatures ranging 4–12 °C and relative humidity of 60–80% [102]. In a geographic and population modeling study conducted in five largest cities in Colombia, the transmission of SARS-CoV-2 seems to be comodulated by temperature and humidity. Their observation revealed a strong reduction of transmission in climates with temperatures above 30 °C and relative humidity below 78% which may comodulate the infectivity of SARS-CoV-2 within respiratory droplets [103].

**Figure 5:**
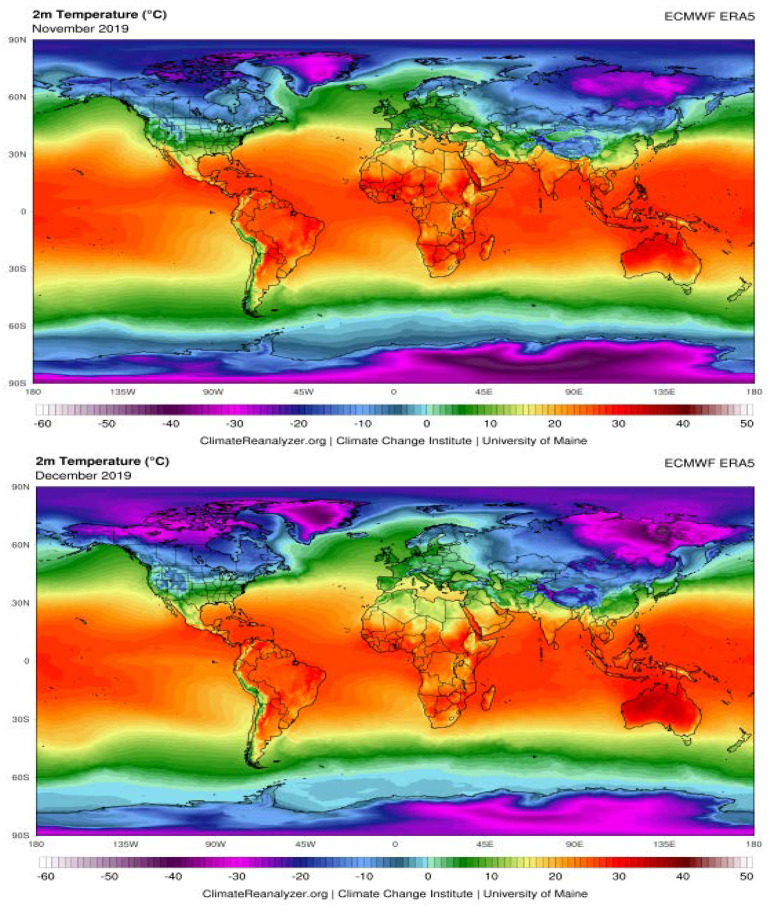

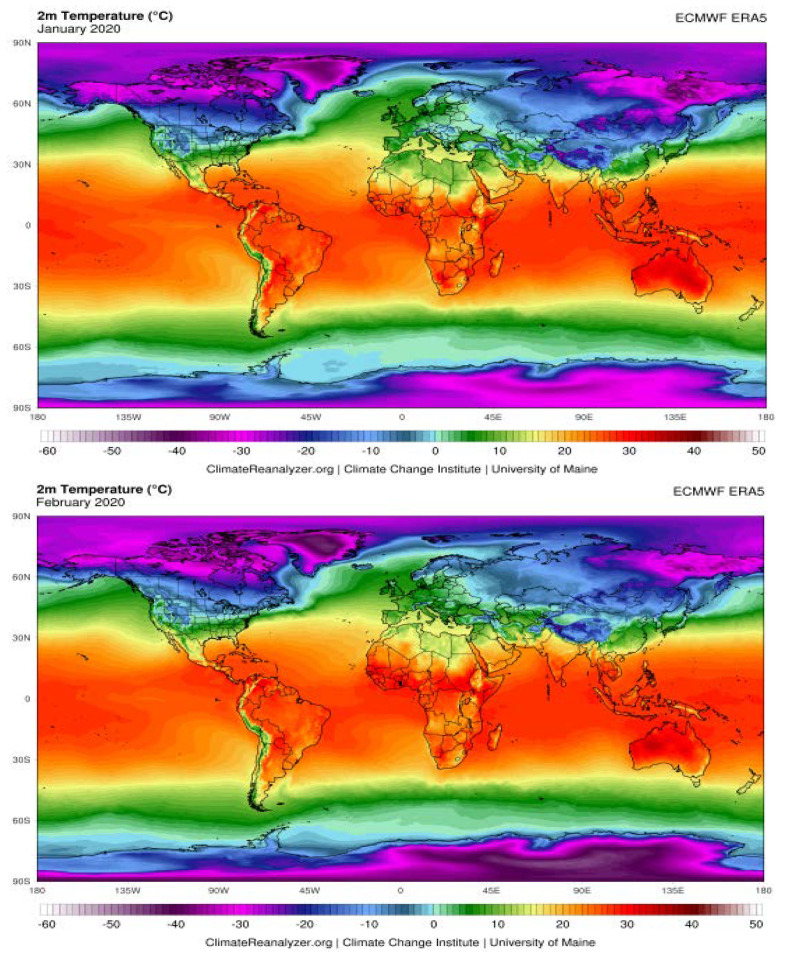
Monthly temperature (degree C) reanalysis maps using ECMWF dataset of all the world. The temperatures at 2-m height, obtained from ERA-interim datasets (https://climatereanalyzer.org/), have been processed to extract monthly means maps for the period November 2019 to February 2020. ERA-interim is a global reanalysis of recorded climate observations over the past 3.5 decades. It is presented as a gridded data set at approximately 0.7 degrees spatial resolution and 37 atmospheric levels. ERA-interim is produced by the European Center for Medium-Range Weather Forecasts (ECMWF) (https://climatereanalyzer.org/).

Overall, these meteorological analyses support that the combination of temperature and humidity could represent a direct influence on the transmission of the COVID-19. It can be assumed that the arrival of summer and rainy season in the NH can effectively reduce the transmission of the COVID-19. The distribution of COVID-19 across different longitudes and latitudes with a range of temperatures and humidity may help to predict the prevalence of this disease in terms of environmental characteristics. This could lead to a better understanding of how the virus spreads around the world (Figure 5). It should be noted that apart from the capability of SARS-CoV-2 to persist on environmental surfaces under favorable atmospheric conditions, the duration of its persistence may be affected by temperature and humidity. However, caution is needed when considering the implications of these findings, which may be subject to confounding. Although warmer climates may slow the spread of SARS-CoV-2, relying on weather changes alone to slow the transmission of COVID-19 are unlikely to be enough. However, using this type of dataset and climate analysis modeling is possible to identify areas that are most likely to be at risk of significant COVID-19 cases in the future and serve as an alarm signal to various government departments and agencies to adopt the necessary measures to prevent virus spread [104]. Moreover, more data are gathering around the world due to the change of the season and all authors agree that the association between temperature/humidity and SARS-CoV-2 is an appreciable hypothesis, but not a certainty yet.

## 4. Clinical Presentations

**Figure 6:**
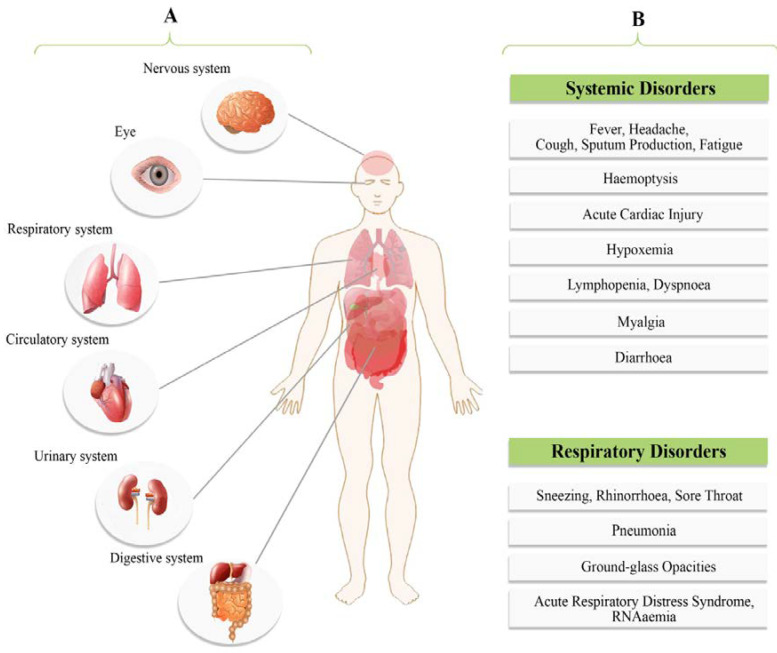
Organ involvement confirmed by clinical features or bioptic sampling in COVID-19 patients (**A**). Table describing main observed disorders (**B**).

The most common symptoms of patients at onset of COVID-19 disease are defined as fever, dry cough, fatigue and less often, symptoms of sputum production, headache, sore throat, myalgia; hemoptysis, dyspnea, diarrhea and lymphopenia were also observed [15,24,28] (Figure 6). The spectrum of clinical features of COVID-19 has been found ranging from an asymptomatic state to severe respiratory failure and multiorgan dysfunction [24,76]. Symptomatic people are considered to be more contagious, similar to most viral-related respiratory diseases. However, individuals who remain asymptomatic may also transmit the virus and cases infected by an asymptomatic individual in the prodrome period of COVID-19 have also been reported [76]. Asymptomatic infections can occur because of weakened immune responses and subclinical manifestations or also because the virus is waiting for opportunities to invade and reproduce. A recent study has shown that a viral load detected in an asymptomatic patient was just like to the one observed in symptomatic patients, indicating the capability of transmission in asymptomatic patients [74]. According to disease presentation, COVID-19 can be classified as mild, moderate, severe and critical (Table 4) [57,76,105].

**Figure 7:**
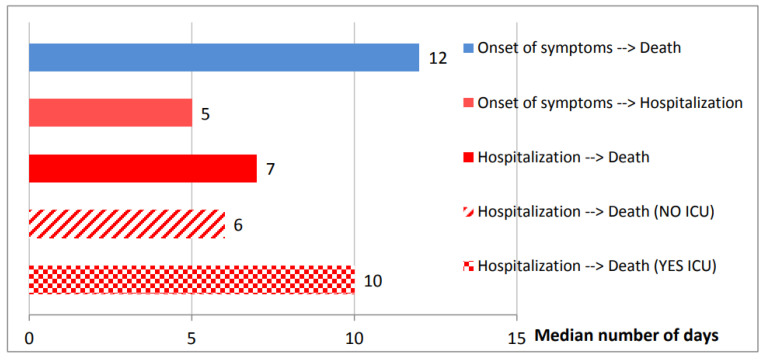
Median times, in days, from the onset of symptoms to death, to hospitalization, from hospitalization to death with and without intensive care unit (ICU)-admittance (Report based on available data on July 9th, 2020 collected from Istituto Superiore di Sanità, ISS).

The symptoms of SARS-CoV-2 infection appear after an incubation period of 1 to 14 days, similar to those of SARS- and MERS-CoV infections (median approximately 5.2 days in different studies) and 95% of patients are likely to experience symptoms within 12.5 days from contact [24,106,107,108]. However, in an asymptomatic carrier the incubation period was 19 days, complicating the challenge to contain the outbreak [109]. The median time between onset of symptoms and dyspnea is 5 days, 7 days for hospitalization and 8 days for acute respiratory distress syndrome (ARDS) (Figure 7) [24] (https://www.epicentro.iss.it/coronavirus/sars-cov-2). Patients at this stage in intensive care unit (ICU) with quarantine facilities may require mechanical ventilation. Moreover, bacterial infections can cause a secondary pneumonia [108]. In addition, the period from the beginning of COVID-19 symptoms to death varied between 6 and 41 days with an average of 14 days [110]. This period depends on immune system status and the patient’s age, being shorter in 70-year-old subjects compared with those younger [78,110]. In people with compromised immune systems and in elderly patients with underlying health problems, SARS-CoV-2 is able to infect the lower respiratory tract leading to severe pneumonia [111]. In 25–30% of patients presenting acute lung injury, shock, ARDS and acute kidney injury, ICU admission was absolutely required [24]. Recovery started within the 2nd or 3rd weeks with the median duration of hospitalization of 10 days. The virus appears to be more fatal in individuals with underlying co-morbidities (50–75% of fatal cases) [24,111]. Available dataset was obtained from Italian Istituto Superiore di Sanità (ISS) on 34,026 patients dying in-hospitals (Figure 7). The mean number of diseases was 3.3 (median 3, SD 1.9). Overall, 4.0% of the reported cases has no co-morbidities, 14.0% with a single comorbidity, 20.6% with 2 and 61.4% with 3 or more (https://www.epicentro.iss.it/coronavirus/sars-cov-2).

SARS-CoV-2 infections revealed some unique clinical characteristics that include targeting the lower airway which is evident by symptoms of upper respiratory tract including rhinorrhoea, sneezing and sore throat [78]. Chest computed tomography (CT) scans revealed pneumonia in most SARS-CoV-2 infected patients and several cases showed an infiltrate in the upper lung lobe, which is related to increasing dyspnea with hypoxemia [28,78,112]. Table 4 describes the full picture of COVID-19 clinical manifestation. Atypical symptoms include RNAemia, acute cardiac injury, ARDS and grand-glass opacities that lead to death [113]. It should be noted that COVID-19 manifestations such as fever, dyspnea, dry cough and bilateral ground-glass opacities in chest CT scans are similar to the previous *Betacoronavirus*-related diseases [113,114]. Although gastrointestinal symptoms such as diarrhea were reported in SARS-CoV-2 infected patients, the similar gastrointestinal distress occurred in only a small percentage of MERS-CoV or SARS-CoV patients (Figure 6) [78]. It was shown that severe cases were characterized by an increased inflammation due to both systemic and localized immune response activation [78,115]. Higher leukocyte numbers, significantly high blood concentrations of cytokines and chemokines were noted in these cases [28,78]. Hence, it is now accepted that high levels of proinflammatory cytokines could worsen the prognosis [28,113].

## 5. Diagnosis

COVID-19 clinical evaluation is focused mainly on epidemiological data, clinical symptoms and clinical and laboratory tests. Although the scenario is continually changing, several approaches were selected as standard laboratory methods for COVID-19 diagnosis. Lab tests, differently from clinical-based analyses, immediately reveal SARS-CoV-2 infected patients. This was particularly important for diagnosis due to the difficulties in detecting specific clinical signs and symptoms in COVID-19 patients. Moreover, atypical manifestations revealed by pulmonary imaging [116] and the huge number of different clinical signs and symptoms forced the development of molecular-based laboratory tests [117,118]. Lastly, the analysis of personal history of each patient played a fundamental role in COVID-19 diagnosis and up to now is considered one of the key information for detecting infected patients also in the early phases of infection. Therefore, the epidemiological history together with clinical and laboratory tests are all required for the diagnosis of COVID-19. A detailed description focused on clinical diagnostic methods was reviewed by Taisheng Li [119]. Herein, we present an updated overview of the principal techniques used for COVID-19 diagnosis.

### 5.1. Nucleic Acid Detection Technology

High-throughput sequencing and real time quantitative polymerase chain reaction (RT-qPCR) are the best nucleic acid detection techniques for SARS-CoV-2. However, in clinical diagnosis, the application of high-throughput sequencing technology is limited due to high cost and its equipment dependency [114,120,121]. Moreover, to speed up the development of standardized analytic kits for diagnostic application, the quantification of viral load was not considered. Therefore, the RT-PCR method was chosen as the gold standard for the detection of SARS-CoV-2 infections from the commonly used samples such as naso- and oropharyngeal swabs [106,107,121,122]. This molecular method relies on the amplification of up to three SARS-CoV-2 specific targets including the RNA-dependent RNA polymerase (*RdRp*), *E* and *N* genes [121]. The WHO has released numerous RT-PCR protocols for the detection of SARS-CoV-2 RNA at https://www.who.int/emergencies/diseases/novel-coronavirus-2019/technical-guidance/laboratory-guidance (Accessed March 15, 2020). Three of those protocols are mentioned below. The US centers for disease control and prevention (CDC) developed an RT-PCR that includes three sets of oligonucleotide primers and probes recognizing three regions of the virus *N* gene (named N1, N2 and N3) and an additional primer/probe set to detect the human *RNase P* gene (RP) representing an internal control for RNA extraction and retro-transcription. Moreover, the positive control consisting in retro-transcribed viral RNA is also available at CDC. To report the positivity for SARS-CoV-2 two out of three N regions must be positive. The Chinese Center for Disease Control and Prevention (China CDC) recommends the use of specific primers and probes targeting the ORF1ab and *N* gene regions for SARS-CoV-2 detection by RT-PCR [123]. The positivity is confirmed when both targets are detected. Available online: http://ivdc.chinacdc.cn/kyjz/202001/t20200121_211337.html (accessed on 21 January 2020).

Overall, the WHO summarized all the primer pairs and probes that can be used to detect SARS-CoV-2 in clinical specimens with the description of RT-PCR settings and the specificity. Apart from the possibility to perform the RT-PCR in house using the selected primer pairs and probes, several ready to use kits were developed for RT-PCR performing. One of the most used is the Allplex 2019-nCoV (Seegene, Seoul, South Korea) which includes three different viral targets and a positive control [124]. Another example is represented by the BGI’s real-time fluorescent RT-PCR Kit for detecting SARS-CoV-2 that includes one SARS-CoV-2 specific target and an internal control of the reaction (BGI, Cambridge, MA, USA). Both companies declared a sensibility of 100–150 viral copies per mL and a high specificity that excludes most respiratory tract viral and bacterial pathogens. The recommended samples for both in-house and ready-to-use RT-PCR kits include upper and lower respiratory specimens such as throat, nasal nasopharyngeal (NP) and/or oropharyngeal (OP) swabs, lower respiratory tract aspirates, sputum, bronchoalveolar lavage (BAL) fluid and nasopharyngeal wash/aspirate or nasal aspirate. It was observed that samples of the lower respiratory tract provide the higher viral loads [74]. On the other hand, it was shown that in the early stage of infection, the positive rate of RT-PCR was reported to be about 60% for throat swab samples [125]. Indeed, although being the gold standard, the RT-PCR presents some drawbacks. One of the most important is related to the sensibility because it was extensively reported that in the presence of low viral load this technique fails in detecting viral genome leading to false negative results [126]. Due to this problem, clinical governance as well as kit troubleshooting indicate to retest all the samples showing only single positive target along with patient resampling. To this respect, it should be underlined that operator skills or sampling sources can profoundly affect RT-PCR testing results [22]. Finally, during this pandemic several microbiologic labs worldwide are experiencing scarce availability of RNA extraction as well as ready-to-use RT-PCR kits increasing the timing of diagnosis confirmation through molecular approaches. Very recently, it was reported that the Allplex 2019-nCoV and the RealStar SARS-CoV-2 RT-PCR kits can amplify the target genes bypassing the RNA extraction step for a faster diagnosis [127].

### 5.2. CT scans and Serology Methods

Although RT-PCR is specific for the diagnosis of COVID-19, its false-negative rate cannot be overlooked due to the severe consequences of missed diagnosis. Clinicians have demonstrated the usefulness of CT and chest radiography for the diagnosis of COVID-19 associated pneumonia [128]. Moreover, the ability of radiologists to diagnose COVID-19 pneumonia from chest CT evaluations has been reported to be very high [129]. Then a combination between RT-PCR and CT imaging represents the best approach for the correct COVID-19 diagnosis. In particular, for early detection and assessment of disease severity, the high-resolution CT (HRCT) of the chest is considered necessary [130,131]. One study analyzed the consistency and diagnostic value of RT-PCR test compared with chest CT in 1014 patients with suspected SARS-CoV-2 infection. Findings indicated that the chest CT sensitivity in suspected patients was 75% based on negative RT-PCR results and 97% based on positive RT-PCR results [132]. Moreover, Salehi et al. confirmed the higher sensibility of pulmonary imaging with respect to RT-PCR for COVID-19 diagnosis and showed a positive correlation between specific CT findings with the different stages of the disease and its severity [116]. The collection of numerous CT images has opened the possibility to build a database of pulmonary images from COVID-19 patients. Interestingly, the recent progress in integrating artificial intelligence (AI) with computer-aided design (CAD) software for diagnostic imaging revealed that AI could be used to support disease diagnosis [133,134]. Ito et al. reviewed the literature on the use of AI for lung diagnostic imaging of COVID-19 patients. Among the 15 selected studies, 11 used AI for CT and 4 used AI for chest radiography. The number of datasets ranged from 106 to 5941, with sensitivities ranging from 67–100% and specificities ranging from 81–100% for prediction of COVID-19 pneumonia. This study revealed the usefulness of AI approach to support the diagnosis of COVID-19, but also for future emerging diseases [134]. All the collected knowledge on lung lesions revealed some characteristic CT findings of COVID-19 pneumonia: the pulmonary ground-glass opacities in a peripheral distribution and the consolidation referring to an increase in pulmonary parenchymal density [135,136,137]. However, chest CT manifestations can vary in different patients and stages of infection, highlighting certain shortcomings of this approach. Apart from atypical manifestation that cannot be recognized by radiologists, several lung images are common in viral pneumonia leading to misdiagnosis [138]. Soon after the beginning of SARS-CoV-2 spreading, infected patients underwent antibody research for both basic research and clinical applications. One of the first studies reported the seroconversion of 100% of infected patients (*n* = 285) within 19 days after symptom onset. Seroconversion for IgM and IgG occurred simultaneously or sequentially and both immunoglobulins titers plateaued within 6 days after seroconversion. Importantly, the application of serology testing in surveillance in a cluster of 164 close contacts of COVID-19 patients identified 4.6% of positive patients showing negative RT-PCR results [139]. Hence, several studies underlined the recommended usage of serology to promote the detection of SARS-CoV-2 infections where NP swab specimens were improperly collected, molecular assays were unsatisfactorily carried out and for determining asymptomatic infections [122]. Based on these data, several companies developed kits for IgM/IgG testing showing a high detection rate of infected patients. Basically, there are two different testing methods: the rapid IgG-IgM test and the classical enzyme-linked immunosorbent assay (ELISA)-based test. The rapid test consists in a lateral flow qualitative immunoassay on a strip to detect the presence of both anti-SARS-CoV-2-IgM and anti-SARS-CoV-2-IgG in human specimens such as whole blood, serum and plasma. This IgG-and IgM-combined antibody test kit has a sensitivity of 88.66% and specificity of 90.63%. Results are obtained in 15 min leading to its useful application as point-of-care testing and in supporting RT-PCR-based diagnostic [140]. On the other hand, several ELISA-based kits are now commercially available, and their sensitivity and specificity were compared showing an overall high specificity, but a variable sensibility [141]. Differently from the rapid tests, the ELISA-based test should be performed on serum or plasma samples collected from venous sampling. Interestingly, the authors showed the neutralizing capacity of SARS-CoV-2 specific antibodies on Caco-2 cells directly incubating the sera from patients with the cell monolayers [141]. This assay is extremely important for the plasma-based therapies that are successfully used to treat seriously ill patients (see below). Finally, recently published papers described the seroconversion of COVID-19 patients including the evaluation of IgA that seems high in the early stages of infection (about 4 days’ post symptom development) [142,143]. Another interesting application of antibody detection is represented by the fluorescence immuno-chromatographic assay for the detection of SARS-CoV-2 nucleocapsid protein in human specimens such as NP swab [144]. It shows the fastness of rapid tests (results in 10 min), the possibility to use the same type of sample that is commonly used for RT-PCR-based diagnosis and high sensibility (detection of the nucleoprotein in all positive samples tested). Although these methods were suggested for COVID-19 diagnosis, the extent of antibodies production by infected patients is greatly variable. Moreover, the delay of antibodies production with respect to the onset of symptoms affects the use of this approach for diagnosis. Vice versa, it is reported that several governments, included Italy, are using serologic test for population screening to assess the proportion of people that have developed an immunological response against SARS-CoV-2 (http://www.salute.gov.it/portale/nuovocoronavirus). This screening will help also to detect asymptomatic and/or paucisymptomatic subjects.

## 6. SARS-CoV-2 Therapeutics Strategies

The rapid spread of SARS-CoV-2 raises an urgent requirement for effective therapeutic strategies against COVID-19. Although many efforts have been intended to develop vaccines against HCoVs infections in recent years, there is no official and effective treatment against SARS-CoV-2. However, different considerable options have been applied for possible vaccine validity, efficacy and safety along with speeding up other ongoing searches to discover valuable modalities for dealing with the emerging COVID-19 [12,145,146,147,148].

**Figure 8:**
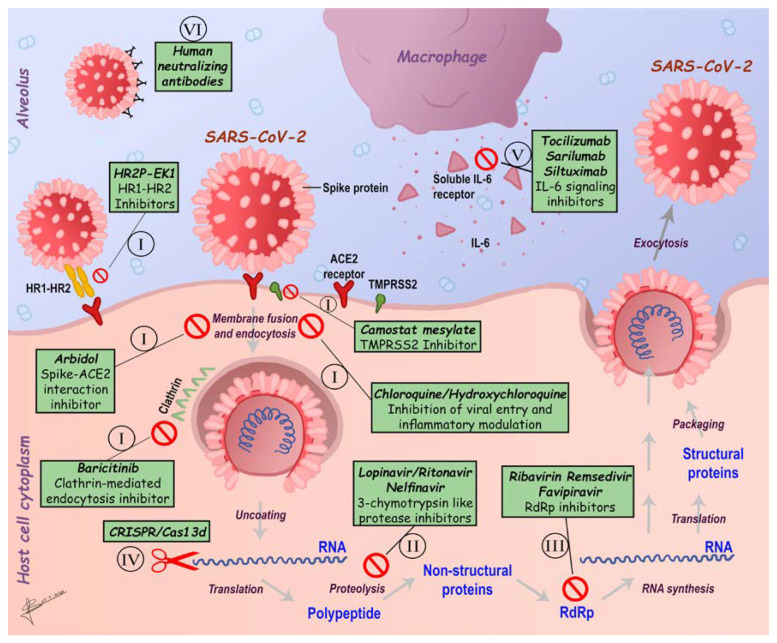
Schematic representation of SARS-CoV-2 infection and virus-induced human immune system response. Proposed drugs directed both towards specific SARS-CoV-2 molecular targets and biologic processes are highlighted: inhibitors of SARS-CoV-2 fusion/entry targeting ACE2 receptor, spike protein, TMPRSS2 or HR1 and HR2 epitopes and clathrin-mediated endocytosis (I); molecules against SARS-CoV-2 main protease (II); molecules against viral genome replication (III); CRISPR technologies targeting SARS-CoV-2 RNA genome (IV); modulators of SARS-CoV-2 induced inflammatory response (V) and human neutralizing antibodies (VI). ACE2, angiotensin-converting enzyme 2; TMPRSS2, type 2 transmembrane serine proteases; RdRp, RNA-dependent RNA polymerase; HR1, heptad repeat 1; HR2, heptad repeat 2; HR2P, heptad repeat 2-derived peptides; EK1, a modified OC43-HR2P peptide. Adapted from [223].

Most of the drugs that are being used to cope with COVID-19 epidemic are directed towards specific viral molecular targets and biologic processes through which the virus spreads damaging the host. In line, all available experimental therapies for COVID-19 management are based on previous experiences in treating SARS-CoV and MERS-CoV infections, such as inhibitors of SARS-CoV-2 fusion/entry/replication, anti-viral agents against main viral proteases, regulators of SARS-CoV-2 induced host inflammatory response and direct administration of human monoclonal antibodies (mAbs) (Figure 8) [149]. Apart from all these possible therapeutic approaches, it has been reported that the Chinese medicine products, as Lianhuaqingwen and ShuFeng JieDu capsules may be helpful for SARS-CoV-2 treatment [12,150]. Indeed, this product is mainly used to treat upper respiratory tract infections such as the flu, swelling and pain in the throat, mumps and strep throat [151,152]. Moreover, four COVID-19 cases have been described to gain improvement after taking combined Chinese and Western medicine [153]. Notably, encouraging progress in deciphering SARS-CoV-2 genome will lead to new potential therapeutic targets. Likewise, more prospective, rigorous population studies are urgently required to confirm the therapeutic effect as well as the safety of new potential therapeutic strategies in order to further implement robust preventive and control measures against SARS-CoV-2 spread.

### 6.1. Inhibitors of SARS-CoV-2 Fusion/Entry

As outlined above, multiple strategies are aimed at developing CoVs vaccines, most of which are headed for the surface-exposed spike (S protein) glycoprotein as the major virus–host cell membrane interactor. To this aim, vaccines under study are based on full-length S protein, S1-RBD, expression of virus-like particles (VLP), DNA or viral vectors [42,154,155,156,157,158]. As outlined above, the S1 includes the RBD that interacts with its host cell receptor, ACE2, whereas the S2 mediates fusion between the virus and host cell membranes promoting the entry and subsequent replication of the viral RNA into the cytoplasm [158]. The ACE2 receptor, as a specific biologic target for vaccine development, is under study in a controlled pilot clinical trial to investigate the effect of recombinant human ACE2 (rhACE2; GSK2586881) in patients with severe COVID-19 (NCT04287686) (Figure 8I) [159,160]. Vice versa, both recombinant proteins containing RBD and the recombinant vectors encoding RBD can be used to generate the effective SARS-CoV vaccines given the capability of this domain to induce neutralizing antibody [156]. Indeed, the first available SARS-CoV-specific human monoclonal antibody with neutralizing activity against SARS-CoV, named CR3022, was found to bind potently to SARS-CoV-2 RBD, in agreement with the high homology shared by this domain with SARS-CoV homolog [161]. However, it must be taken into account that more than 85% of the RBD antibody epitopes in SARS-CoV-2 show implicit noticeable changes, indicating the necessity to develop more specific monoclonal antibodies for SARS-CoV-2 [162].

Angiotensin receptor blockers (ARBs), such as losartan, valsartan, telmisartan, usually assumed for treating high blood pressure, heart and kidney failure in people with diabetes, have been recently proposed as a novel therapeutic approach to block SARS-CoV-2 RBD binding to ACE2- expressing cells binding, similarly to ACE inhibitors [163].

Additional targetable epitopes that should be considered are the heptad repeat 1 (HR1) and heptad repeat 2 (HR2) in SARS-CoV-2 S protein. In fact, the HR2-derived peptides (HR2P) and EK1 (a modified OC43-HR2P peptide), exhibit effective fusion inhibitory activity towards SARS-CoV-2, suggesting a promising strategy in treating SARS-CoV-2 infection, although further studies are required to strengthen these hypotheses (Figure 8I) [164,165].

Lately, immuno-informatics have been employed to identify significant cytotoxic T lymphocyte (CTL) and B-cell epitopes in SARS-CoV-2 S protein, such as the nucleocapsid (N) protein as well as the potential B cell epitopes of the E protein of MERS-CoV as likely immunoprotective targets [166,167].

Reverse genetic strategies have been successfully used in live-attenuated vaccines to inactivate the exonuclease effects of non-structural protein 14 (nsp14) or to wipe out the envelope protein in SARS [154]. A recent study also revealed that the invasion process requires the priming of the S protein which is facilitated by the host cell produced serine protease TMPRSS211. The clinically demonstrated serine protease TMPRSS2 inhibitor Camostat mesylate, which partially blocks SARS-CoV-2 entry into host cells, was shown to be a good target to significantly reduce pulmonary infection in COVID-19 affected individuals (Figure 8I) [168] Moreover, it has been suggested that coronavirus entry also involves pH and receptor-dependent endocytosis [169,170]; thus, targeting endocytosis may be another assessable option for fighting SARS-CoV-2 (Figure 8I). In this view, throughout AI technology, a group of approved drugs, such as the Janus kinase (JAK) inhibitor baricitinib [171] targeting the AP-2-associated protein kinase 1 (AAK1) regulating clathrin-mediated endocytosis, has been developed (Figure 8I) [172]. Furthermore, other drugs such as arbidol (ChiCTR2000029621), a haemagglutinin inhibitor and chloroquine phosphate, a traditional antimalarial drug, have been added to the National Health Commission of the People’s Republic of China (NHC) guidelines for COVID-19 treatment (Figure 8I) (http://www.nhc.gov.cn). In particular, in vitro studies have demonstrated that chloroquine as well as hydroxychloroquine could impair the endosome-mediated viral entry or later stages of viral replication [173]. Combination of hydroxychloroquine and azithromycin has also been suggested as a valid approach since it showed more rapid resolution of infection than hydroxychloroquine alone [174]; however, the combined use of azithromycin and hydroxychloroquine seems to be associated with at increased risk of arrhythmias. Available online: https://www.acc.org/latest-in-cardiology/articles/2020/03/27/14/00/ventricular-arrhythmia-risk-due-to-hydroxychloroquine-azithromycin-treatment-for-covid-19 (accessed on 29 March 2020).

### 6.2. Inhibitors of SARS-CoV-2 Main Enzymes

To date, several attempts have also been made in targeting viral main enzymes; in fact, many inhibitory drugs targeting the coronavirus main proteinase 3C-like protease (3CLpro) have been validated in clinical trials (e.g., Lopinavir/Ritonavir; ChiCTR2000029387, ChiCTR2000029468, ChiCTR2000029539) (Figure 8II) [175]. Moreover, four additional molecules including prulifloxacin, tegobuvir, bictegravir and nelfinavir, detected by high-throughput screening, showed reasonable binding conformations with the viral main protease [176]. Moreover, a recent study by performing a virtual screening using a three-dimensional model of the SARS-CoV-2 3C-like protease (3CL), identified 16 biologic candidates that deserve further consideration. Among these, the antivirals Ledipasvir or Velpatasvir proved to be particularly attracting as therapeutics to combat the new coronavirus showing optimal anti-viral activity and minimal side effects, such as fatigue and headache; also, Epclusa (velpatasvir/sofosbuvir) and Harvoni (ledipasvir/sofosbuvir) are promising antivirals, not only for their effective and synergic inhibitory activities against two viral enzymes, but also for their minimized possibilities to develop resistance [177].

### 6.3. Inhibitors of SARS-CoV-2 Replication

A certain number of clinical trials on antiviral drugs aimed to arrest SARS-CoV-2 replication are currently in progress, such as Remdesivir (NCT04252664, NCT04257656) Favipiravir (ChiCTR2000029600, ChiCTR2000029544) and ASC09 (ChiCTR2000029603) (Figure 8III). Among these, Remdesivir was recently approved for medical use in America and European Union and seems to be the most promising antiviral for fighting SARS-CoV-2 [178] (http://www.who.int), as in vitro studies demonstrated that this molecule, a mono-phosphoramidate prodrug of an adenosine, effectively inhibited SARS-CoV-2 RNA synthesis [179]. Targeting the SARS-CoV-2 RNA genome could, therefore, be another potential strategy. In fact, a CRISPR/Cas13d technology, which is an RNA-guided RNA-targeting CRISPR system, has been employed to specifically chew up SARS-CoV-2 RNA genome. In this system, a Cas13d protein and guide RNAs-containing spacer sequences are used to specifically complement the virus RNA genome (Figure 8IV). Furthermore, RNA genome can be packaged into one adeno-associated virus (AAV) vector, making the CRISPR/Cas13d system more efficient for virus elimination and resistance prevention, taking into account that AAV has serotypes highly specific to the lung, the main organ infected by SARS-CoV-2 [180].

### 6.4. Modulators of SARS-CoV-2 Induced Inflammatory Response

In addition to antiviral therapy, a new treatment strategy having a significant impact on clinical outcomes is utmost required. Immunomodulatory therapy to downregulate the cytokine storm may provide great benefit to the treatment of COVID-19. Recently, researchers focused on targeting specific molecular markers involved in inflammatory cytokines-receptors interactions, their correlation in health and disease and drugs in use that can activate or block their actions. A higher concentration of cytokines has been found in the plasma from COVID-19 patients in ICU compared with the ones from non-ICU COVID-19 patients, suggesting that the cytokine storm could be linked to the severity of the disease [113]. Corticosteroids are among the most commonly used drugs for immunomodulatory therapy of infectious diseases. However, the use of corticosteroids in the treatment of COVID-19 can cause host immune suppression and delay of viral clearance. A recent study on 201 patients with ARDS showed that treatment with methylprednisolone decreased the risk of death (hazard ratio 0.38, 95% confidence interval 0.20–0.72). These findings indicate that using corticosteroids does not influence viral clearance time, length of hospital stays or duration of symptoms in patients with mild COVID-19 [181]. Thus, the use of corticosteroids is considered beneficial in severe cases of COVID-19 (especially in patients with ARDS), but not in mild cases. Accordingly, a recent retrospective study showed the potential benefits from low-dose corticosteroids treatment in a subset of critically SARS-CoV-2 patients [182]; these data are in contrast with NHC guidelines that highlight that systematic use of corticosteroids is not recommended for these cases, due to their immunosuppressive effects. However, administration of corticosteroids has been indicated for specific reasons such as exacerbation of asthma or chronic obstructive pulmonary disease (COPD), septic shock or severe acute respiratory distress syndrome (ARDS). Further studies are required to find out how and when it is appropriate the use of corticosteroids for COVID-19, as there are no available data on the benefits of corticosteroid treatment in SARS-CoV or MERS infection [183].

Apart from corticosteroids, IL-6 pathway inhibitors such as sarilumab, siltuximab and tocilizumab have been proposed as experimental approach considering the increased IL-6 levels that have been observed in patients with severe COVID-19 [184]. Tocilizumab is a recombinant, humanized monoclonal antibody commonly used for treating patients with rheumatoid arthritis, lupus and psoriasis that binds to IL-6 receptors blocking FcR activation; in COVID-19 patients, Tocilizumab could reduce SARS-CoV-2-induced inflammatory responses [185]. Accordingly, several case reports have referred positive outcomes regarding Tocilizumab [113,186,187,188,189,190], but clinical impact of Tocilizumab on COVID-19 patients as an approved clinical approach has not been evaluated yet. In line, to further investigate the efficacy and safety of Tocilizumab in patients with COVID-19, a controlled clinical trial is now under way (ChiCTR2000029765) (Figure 8V). Overall, the combination of an immunomodulatory agent to reduce the cytokine storm with an antiviral agent may give physicians more time to provide supportive treatment for patients with COVID-19.

### 6.5. Passive Immunization

At the time of writing this review, due to the lack of a specific available therapy, plasma from convalescent patients containing specific antibodies has been proposed as a principal treatment [190,191], for patients in rapid disease progression, severe or critical conditions (Figure 8VI). In a recent retrospective study, one dose (200 mL) of convalescent plasma (CP) collected from 10 severe adult cases has been reported to be tolerated; thus, increasing or maintaining high level of neutralizing antibodies broke down the viral load in seven days, improve clinical symptoms and paraclinical criteria within three days and lung lesions were found to be differently absorbed on radiological examination within seven days [192]. Therefore, being CP a promising rescue option for severe COVID-19, several clinical trials (ChiCTR2000030010, ChiCTR2000030179, and ChiCTR2000030381) are in progress to investigate the efficacy and safeness of CP direct infusion in COVID-19 patients [191]. In addition, combined therapy with mAbs and Remdesivir seems to be an ideal therapeutic option for COVID-19 [193]. Pharmaceuticals companies are now focused on searching for specific and effective mAbs against COVID-19. Taking into account that technologies capable of making fully human antibodies such as human single-chain antibody variable fragments (Hu-scFvs) or humanized-nanobodies (single-domain antibodies, sdAb) able to overpass virus-infected cell membranes (trans bodies) and to interact or interpose with biologic processes required for virus replication are already available [194].

### 6.6. Cell-Based Therapies

A large number of clinical trials regarding cell-based therapies have been started in China during COVID-19 outbreak. Among these, mesenchymal stromal cells (MSCs)-based therapy displayed strong safety profile and possible efficacy in patients with ARDS, according to COVID-19-related clinical studies listed on the WHO’s International Clinical Trials Registry Platform (WHO ICTRP) and National Institutes of Health’s clinical trials.gov databases [195]. Nevertheless, further investigations are required to better understand if these therapies could be effective in treating respiratory virus-induced complications. MSCs have been largely employed in basic research and clinical trials [196,197,198], and their safeness and effectiveness have been extensively documented especially in immune-mediated inflammatory disorders, such as graft-versus-host disease (GVHD) [199] and systemic lupus erythematosus (SLE) [200]. MSCs immunomodulatory and differentiation abilities [201] as well as their competency to produce several cytokine types or to directly interact with immune cells have been already described [202]. Indeed, they are activated by pathogen-associated molecules (PAMPs) such as single or double-stranded RNAs [203,204], priming the immune response during infections. Two clinical investigations of systemic MSC administration in patients with either COVID-19 or avian influenza A (H7N9) have been recently published [205,206]. The first one, a single-center MSC transplantation pilot study, was aimed at exploring MSCs therapeutic potentiality in patients with COVID-19 pneumonia and conducted at the You’an Hospital in Beijing, China, from 23 Jan 2020 to 16 Feb 2020 (ChiCTR2000029990). Seven patients with COVID-19 pneumonia, SARS-CoV-2 RNA positive, with different degrees of severity, including one critically ill requiring ICU care were enrolled and monitored for 14 days after MSC injection. A significant improvement of pulmonary function and symptoms were observed two days after MSC transplantation characterized by an increase of peripheral lymphocytes and of the anti-inflammatory IL-10 levels and a decrease of the C-reactive protein and TNF-α amounts [205]. Moreover, an increment of the CD14 + CD11c + CD11b^mid^ regulatory dendritic cell (DC) population and a decrease of cytokine-secreting immune cells such as CXCR3 + CD4 + T cells, CXCR3 + CD8 + T cells, and CXCR3 + NK were detected within 3–6 days in the treated patients compared to the placebo control group [205]. MSCs play a role in attenuating cytokine storm, most importantly, because these cells do not express ACE2 and TMPRSS2 viral receptors are insusceptible of SARS-CoV-2 infection. These observations are in agreement with the knowledge that MSCs induce the maturation of dendritic cells into a novel Jagged-2-dependent regulatory dendritic cell population [207], shifting the Th1/Th2 balance towards Th2. Thus, from these preliminary results, it seems evident that MSCs intravenous transplantation could represent a secure and effective treatment in patients with COVID-19 pneumonia, especially those critical. Indeed, it inhibits the over activation of the immune system and promotes endogenous repair by preventing pulmonary fibrosis and improving both pulmonary microenvironment and lung function [205].

### 6.7. Vaccines

More than 15 potential vaccine candidates for COVID-19 are under development around the world, including inactivated, recombinant subunits, nucleic-acid-based, adenoviral vector, and recombinant influenza viral vector vaccines [208]. Moreover, taking into consideration the strong homologies existing among the various coronavirus strains, it was thought that vaccines acting on other coronaviruses, such the avian live IBV vaccine (strain H) directed towards the chicken CoV IBV, could be a valuable alternative therapeutic strategy [209].

The Coalition for Epidemic Preparedness Innovations (CEPI) recently announced that three programs aimed to develop COVID-19 vaccines, by utilizing established vaccine platforms, have started [210]. In addition, CEPI already financed the company Moderna, Inc. to compare mRNA therapeutics and vaccines, allowing the release of the first batch of mRNA-1273 in February 2020, which is an mRNA vaccine against SARS-CoV-2 ready for phase I study in the United States. Available online: https://investors.modernatx.com/news-releases/news-release-details/moderna-ships-mrna-vaccine-against-novel-coronavirus-mrna-1273 (accessed on 24 February 2020).

More recently, scientists from the University of Pittsburgh have announced a potential vaccine against SARS-CoV-2, delivered throughout a fingertip-sized patch, capable of producing SARS-CoV-2 specific IgG antibodies, sufficient for virus neutralization in mice. This vaccine, called PittCoVacc (acronym of Pittsburgh coronavirus vaccine), is a trimeric recombinant SARS-CoV-2-S1 subunit vaccine delivered intracutaneously by microneedle arrays (MNAs) [211]. Delivering vaccine components to a defined skin microenvironment improves safety by reducing systemic exposure, allowing to reach high vaccine concentrations with a relatively low dose of antigen [212,213]. Furthermore, the skin delivery strategy promotes strong and long-lasting antigen-specific antibody responses due to both the high immunogenicity [214,215,216,217,218] and the redundant immunoregulatory circuits of the skin [217,219,220]. Given the urgent need for COVID-19 vaccines, MNAs strategy seems to be a promising immunization approach against coronavirus infection including SARS, MERS and other emerging infectious diseases.

On April 24, the Oxford ChAdOx1 nCov-19 vaccine was the first in Europe to start human trial stage, with 1110 healthy volunteers enrolled for the tests. Oxford scientists have already employed ChAdOx1 in the past to dispense vaccines against Ebola, Chikungunya, Rift Valley fever and, above all, MERS. ChAdOx1, a chimpanzee-derived adenovirus vector, has been employed to deliver the full-length MERS spike gene and shown to induce large amounts of neutralizing antibodies against MERS in a mouse model [221,222]. Therefore, the modified ChAdOx1 vaccine, carrying the SARS-CoV-2 spike gene is under human trial stage. On April 30, the University of Oxford has announced a collaboration with the UK-based global biopharmaceutical company AstraZeneca for further development, large-scale production and potential delivery of the COVID-19 vaccine candidate. Available online: https://www.ovg.ox.ac.uk/news/landmark-partnership-announced-for-development-of-covid-19-vaccine (accessed on 30 April 2020). Since ChAdOx technology is already available and formerly tested in humans for other vaccines, Phase III will consist in administering vaccine to volunteers following them into their regular environments to ensure that these subjects actually become immune to the disease up to three years. If trials succeed, Oxford researchers have proposed to complete testing throughout ring vaccination, namely delivering vaccine to members of the first circle of contacts of COVID-19 positive people and then to evaluate if the virus spreads to the second circle, as was previously done during the 2018 Ebola epidemic in the Democratic Republic of the Congo.

Overall, a joint effort headed to apply both already consolidate and innovative approaches, such as AI to facilitate drug discovery, will be required to develop a broad-spectrum antiviral drugs and vaccines towards existing and potential future coronavirus infections to prevent another highly pathogenic virus epidemic. Moreover, continuous collaboration in basic and clinical studies will improve the discovery of new antiviral drugs with therapeutic potentials, decrease the time for drug release on the market and make them affordable for all countries. Furthermore, vaccine delivery strategies and cell-based therapies benefit from the significant progresses made by recombinant DNA technologies combined with emerging biotechnology and bioengineering methodologies. Thus, these approaches can speed up the development and set up of new vaccines and clinical therapies to fight against novel pathogens to protect public health all over the world.

## 7. Conclusions

This study represents a holistic picture of the current investigations in response to the outbreak of COVID-19. The current pandemic is obviously an international public health problem and it remains a challenging task to fight the SARS-CoV-2 of unknown origin and mysterious biologic features. Lesson from the previous two pandemics, MERS and SARS outbreaks, provide valuable insights about how to manage the current pandemic and provide a reference for future studies to combat disease progression. Despite SARS-CoV-2 rapid transmission, the scale up country readiness, speedy response teams and the capacity of all laboratories are reducing the spread of the virus as well as its mortality rate. As the pandemic is still ongoing and expanding, further studies on all aspects of the disease are needed to better understand the infection, beneficial treatments and development of vaccines. Nevertheless, this pandemic, together with the previous ones, have taught us in the harshest possible way that the entire scientific community must be vigilant and ready to advice appropriate containment and screening measures to avoid the spread of any future emerging pathogen.

## Figures and Tables

**Table 1:** Host factors(s) involved in SARS-CoV, MERS-CoV and the SARS-CoV-2 replications [4,15].

Replication Stage	Host Receptor	Virus	Function
**Attachment and entry**	Human angiotensin-converting enzyme 2 (ACE2)	SARS-CoV and most probably SARS-CoV-2	Cellular receptor
	Human dipeptidyl peptidase 4 (DPP4 or CD26)	MERS-CoV	Cellular receptor
	Cathepsin L	SARS-CoV	Cleave and activate S protein
	Furin	MERS-CoV	Cleave and activate S protein
	TMPRSS11D	SARS-CoV	Cleave and activate S protein
	IFITM	SARS-CoV, MERS-CoV	Restrict virus entry
**Genome replication and transcription**	GSK3	SARS-CoV	Phosphorylate N protein and facilitate viral replication
	hnRNPA1	SARS-CoV	Regulate viral RNA synthesis
**Translation of structural proteins**	N-linked glycosylation enzymes	SARS-CoV	Modify S and M protein; N-linked glycosylation of the S protein facilitates lectin-mediated virion attachment and constitutes some neutralizing epitopes
	ER chaperones	SARS-CoV	Proper folding and maturation of S protein

**Table 2:** Comparison of main features among SARS-CoV, MERS-CoV and SARS-CoV-2 [24,76,86,88,93,94,95].

Virus	Receptor	Primary Host	Intermediate Host	Incubation Period	Number of Cases	Number of Deaths	Fatality	R0
SARS-CoV	ACE2	Bats	Civets and raccoon dogs	between 2 and 10 days and up to 14 days	8098	916	~10%	2–5
MERS-CoV	DPP4 (CD26)	Bats	Camels	between 2 and 14 days	2494	858	~35%	2–5
SARS-CoV-2	ACE2	Bats	Manis javanica, others?	Current estimates between 2 and 10 days and up to 14 days	17,889,134 August 3rd, 2020	Over 686,145 August 3rd, 2020	~3.8% August 3rd, 2020	2–6.47

**Table 3:** Average humidity and temperature in 10 different cities in Europe and North Africa between November 2019 to March 2020. The first five cities represent significant communities where transmission of COVID-19 was reported, whereas the second 5 cities are expected to be less exposed to COVID-19 due to different weather conditions.

City	Average Humidity (%)					Temperature (°C)				
	Nov.	Dec.	Jan.	Feb.	Mar.	Nov.	Dec.	Jan.	Feb.	Mar.
Rome	71	65	66	63	63	15	1	11	13	14
Paris	78	76	79	74	66	9	8	8	9	9
Madrid	70	67	68	63	61	10	10	9	13	12
Milan	77	74	69	58	62	11	8	7	11	11
Lisbon	75	74	77	76	71	15	14	12	15	15
Rabat	72	71	69	72	74	16	17	14	17	16
Algiers	64	62	61	61	66	16	17	15	18	16
Tunis	61	66	72	65	70	17	16	14	15	15
Tripoli	80	83	73	75	71	15	10	8	9	11
Cairo	45	52	55	53	46	25	18	16	18	22

**Table 4:** Clinical manifestations of COVID-19.

Clinical Types	Symptoms
Mild	In 81% of all confirmed COVID-19 cases. Dry cough, mild fever, sore throat, nasal congestion, muscle pain, headache and malaise. Absence of serious symptoms like dyspnea, also the absence of radiograph features. It may rapidly deteriorate into severe or critical cases, non-pneumonia or mild pneumonia.
Moderate	Dry cough, tachypnea and shortness of breath.
Severe	Acute respiratory distress syndrome (ARDS), severe pneumonia, severe dyspnea, sepsis or septic shock, tachypnea (respiratory frequency) ≥ 30/min, blood oxygen saturation (SpO_2_) ≤ 93%, partial pressure of arterial oxygen to fraction of inspired oxygen ratio (PaO_2_/FiO_2_) < 300, and/or lung infiltrates > 50% within 24 to 48 h. Fever can be absent or moderate.
Critical	In 5% of all confirmed COVID-19 cases. Respiratory failure, septic shock, RNAemia, cardiac injury and/or multiple organ dysfunction or failure. Case fatality rate is 49% (higher case fatality rate for patients with preexisting co-morbidities and lower-case fatality rate (0.9%) for patients without co-morbidities). Cardiovascular disease (10.5%), diabetes (7.3%), respiratory disease (6.5%), hypertension (6%) and oncological complications (5.6%).

## Abbreviations

The following abbreviations are used in this manuscript: 16 nonstructural polyproteins (nsps 1–16); 2019 novel coronavirus (2019-nCoV); 3-chymotrypsin-like protease (3CLpro); 3C-like protease (3CL); Acute respiratory distress syndrome (ARDS); adeno-associated virus (AAV); angiotensin receptor blockers (ARBs); angiotensin-converting enzyme 2 (ACE2); AP-2-associated protein kinase 1 (AAK1); artificial intelligence (AI); avian infectious bronchitis virus (IBV); basic reproduction number (R0); blood oxygen saturation (SpO2); bronchoalveolar lavage (BAL); centers for disease control and prevention (CDC); Chinese Center for Disease Control and Prevention (China CDC); chronic obstructive pulmonary disease (COPD); Coalition for Epidemic Preparedness Innovations (CEPI); computed tomography (CT); computer-aided design (CAD); convalescent plasma (CP); coronavirus (CoV); cytotoxic T lymphocyte (CTL); dendritic cell (DC); dipeptidyl peptidase 4 (DPP4, also known as CD26); envelope glycoprotein (E); enzyme-linked immunosorbent assay (ELISA); European Center for Medium-Range Weather Forecasts (ECMWF); European cities (ECs); feline infectious peritonitis (FIP); graft versus-host disease (GVHD); hemagglutinin-esterase glycoprotein (HE); heptad repeat 1 (HR1) and heptad repeat 2 (HR2); high-resolution CT (HRCT); HR2-derived peptides (HR2P); EK1 (a modified OC43-HR2P peptide); human coronaviruses (HCoVs); human CoVs (HCoVs); human single-chain antibody variable fragments (Hu-scFvs); humanized-nanobodies (single-domain antibodies, sdAb); intensive care unit (ICU); Istituto Superiore di Sanità (ISS); Janus kinase (JAK); membrane glycoprotein (M); mesenchymal stromal cells (MSCs); microneedle arrays (MNAs); Middle East Respiratory syndrome coronavirus (MERS-CoV); monoclonal antibodies (mAbs); naso-pharyngeal (NP); National Health Commission of the People’s Republic of China (NHC); nonstructural protein 14 (nsp14); Northern Hemisphere (NH); nucleocapsid phosphoprotein (N); open reading frames (ORFs); oro-pharyngeal (OP); papain-like protease (PLpro); partial pressure of arterial oxygen to fraction of inspired oxygen ratio (PaO2/FiO2); pathogen-associated molecules (PAMPs); Pittsburgh coronavirus vaccine (PittCoVacc); polypeptides (pps); porcine epidemic diarrhea virus (PEDV); real time quantitative polymerase chain reaction (RT-qPCR); receptor-binding domain (RBD); recombinant human ACE2 (rhACE2); replication–transcription complex (RTC); RNA-dependent RNA polymerase (*RdRp*); *RNase P* gene (RP); severe acute respiratory syndrome coronavirus (SARS-CoV); single-stranded positive-sense RNA (+ssRNA); Southern Hemisphere (SH); spike glycoprotein (S); subgenomic RNAs (sgRNAs); swine acute diarrhea syndrome coronavirus (SADS-CoV); swine enteric *Alphacoronaviruses* (SeACoVs); systemic lupus erythematosus (SLE); transmissible gastroenteritis virus (TGEV); virus-like particles (VLP); WHO’s International Clinical Trials Registry Platform (WHO ICTRP); World Health Organization (WHO).
